# Therapeutic Drug Monitoring of Infliximab and Adalimumab through Concentration and Anti-Drug Antibodies Assessment; Comparison of Sanquin Diagnostics and Theradiag Assays

**DOI:** 10.3390/antib13030073

**Published:** 2024-09-05

**Authors:** Wim H. M. Vroemen, Shakira S. Agata, Joyce J. B. C. van Beers, Jan G. M. C. Damoiseaux

**Affiliations:** Central Diagnostic Laboratory, Maastricht University Medical Center+, P. Debyelaan 25, 6229HX Maastricht, The Netherlands; wim.vroemen@mumc.nl (W.H.M.V.); s.agata@student.maastrichtuniversity.nl (S.S.A.); joyce.van.beers@mumc.nl (J.J.B.C.v.B.)

**Keywords:** biologics, therapeutic drug management

## Abstract

Background: Therapeutic drug monitoring of biological Tumor Necrosis Factor (TNF)-alpha inhibitors is of critical importance. In this study, the performance of practically advantageous chemiluminescent immunoassays of Theradiag, assessing Infliximab and Adalimumab serum concentrations and anti-drug antibodies (ADA) against these biologics, were compared to the Enzyme-Linked Immuno-Sorbent Assays (ELISAs) from Sanquin Diagnostics. Methods: Leftover serum samples (*n* = 80 for each parameter) from patients treated with Infliximab or Adalimumab were collected. Correlation and agreement analyses for serum concentration and ADAs, respectively, were performed. Both Theradiag ADA assays, an assay targeting both free and bound ADAs and an assay targeting solely free ADAs, were investigated and compared to the Sanquin Diagnostics ADA assay, targeting both free and bound ADAs. Results: Strong positive correlations were observed between the biologic concentration assessment of Infliximab (Spearman’s Rho = 0.91) and Adalimumab (Spearman’s Rho = 0.94). However, there appeared to be significant bias in the Theradiag assay when compared to Sanquin (Infliximab median (Confidence Interval (CI)) = 2.1 (1.7–2.6) µg/mL; Adalimumab median (CI) = 0.8 (0.5–0.9) µg/mL). Agreement analyses showed moderate to good agreement for the Theradiag and Sanquin Diagnostics ADA assays, when detecting both free and bound ADAs, for Infliximab (Cohen’s *k* = 0.717) and Adalimumab (Cohen’s *k* = 0.802). In contrast, the Theradiag ADA assay detecting solely free ADAs had zero to poor agreement for Infliximab (Cohen’s *k* = 0.458) and Adalimumab (Cohen’s *k* = 0.119), respectively. Conclusions: This study demonstrated strong correlations and good agreement between the Theradiag and Sanquin Diagnostics assays measuring Infliximab and Adalimumab serum concentrations and ADAs, both free and bound, against these biologics. Discordance analyses showed significantly decreased drug concentrations in the solely free assays, indicating that the combined detection of free and bound ADAs better aligns with drug levels.

## 1. Introduction

Anti-tumor necrosis factor-alpha (anti-TNF-alpha) agents have permanently modified the landscape of therapeutic options in multiple chronic auto-inflammatory diseases such as inflammatory bowel diseases (IBD), rheumatoid arthritis (RA), and psoriasis [1]. These monoclonal antibodies (mAbs) target TNF-alfa, an inflammatory cytokine abundantly generated in these diseases. The two most established anti-TNF-alpha biologics are Infliximab and Adalimumab. Infliximab is a chimeric mAb consisting of a murine Fab fragment combined with a human Fc fragment and is intravenously administered, while Adalimumab is a completely humanized IgG mAb, which is subcutaneously delivered.

Despite the fact that anti-TNF-alpha biologics have been proven to have significant therapeutic benefits in multiple chronic auto-inflammatory diseases, a significant proportion (up to 70%) of patients unfortunately lose therapeutic response over time due to the formation of anti-drug antibodies (ADAs) [2]. These ADAs can not only reduce drug efficacy by targeting the antigen-binding site of the biologic, therefore neutralizing the agent’s biological activity, but can also cause severe adverse events through the formation of pathogenic immune complexes [2,3,4,5]. Work has been carried out to investigate the effects of the epitopes of anti-drug antibodies against Infliximab [6]. However, it remains to be explored whether there are epitope differences in ADAs, reducing their efficacy, neutralizing therapeutic effects, and allowing the formation of pathogenic immune complexes. Nevertheless, therapeutic drug management, through assessing biologic concentration as well as ADA levels, is of pivotal importance when prescribing these medications [7].

Successful therapeutic drug management requires assays that are able to assess serum drug levels and ADAs accurately. To date, many different methods have been used to develop such assays, all with their own technical (dis)advantages and interference factors [8]. Enzyme-linked immunosorbent assays (ELISAs) are the most commonly used methods to assess biologic concentrations and ADAs, especially in The Netherlands. Despite their many advantages, ELISAs have certain limitations, such as their time-consuming procedure and batch-wise approach, causing low throughput and potentially therapeutic management delays [9]. Recently, Theradiag developed novel chemiluminescent i-Tracker immunoassays, potentially overcoming these limitations.

The goal of this study was to compare the Infliximab and Adalimumab concentration and ADA levels found using the chemiluminescent immunoassays (CLIA) of Theradiag or the ELISAs of Sanquin Diagnostics.

## 2. Methods

### 2.1. Patient Population and Sample Selection

Leftover samples from patients treated with Infliximab or Adalimumab were collected from 2021 to 2023 for concentration assessment and from 2016 to 2023 for ADA assessment. Inclusion required biologic concentration and/or ADA level assessment as part of their routine clinical care. To minimize selection bias and to assess the Theradiag assays across a broad biologic or ADA concentration range, systematic sampling by applying a standard sample interval was performed for the biologic concentration cohorts (Infliximab *n* = 80, Adalimumab *n* = 80), based on the clinically measured Infliximab or Adalimumab Sanquin concentration. For the ADA cohorts (anti-Infliximab *n* = 80, anti-Adalimumab *n* = 80), 10 negative samples were selected, followed by systematic sampling based on Sanquin ADA concentration for the remaining 70 samples. The 320 performed tests involved the samples of 293 individuals. The samples of 27 patients were included in more than 1 of the 4 cohorts.

Ethical approval was not required as the study was non-interventional, all data were completely anonymized and no patients were included who objected against further use of their biological samples. Additionally, the study was performed according to the Code of Conduct for Health Research and the Declaration of Helsinki.

### 2.2. Sample Collection

Serum samples were retrieved by collecting blood samples through venipuncture in BD Vacutainer SST^TM^ II Advance (REF: 367955) gel collection tubes. After the manufacturer recommended 30 min of clotting time, samples were centrifuged at 1885× *g* for 10 min. Subsequently, a serum aliquot (separated from the cells) was directly stored at −20 °C until analysis.

### 2.3. Biologic Concentration and Anti-Drug Antibody (ADA) Assays

Sanquin Diagnostics: Infliximab and Adalimumab concentration and ADA level assessment for routine clinical care were determined using the assays from Sanquin Diagnostics (Amsterdam, The Netherlands). It is important to note that the Sanquin Diagnostics ADA assays detected both free and bound ADAs against Infliximab or Adalimumab. The characteristics of these assays are described in Table 1.

Theradiag: The i-Tracker CLIAs of Theradiag (Croissy-Beaubourg, France), measured on the IDS-iSYS multi-discipline automated system analyzer, are the assays under investigation. In addition to the Infliximab and Adalimumab concentration assays, Theradiag has two types of ADA assays, namely commercially available assays detecting exclusively free ADAs and research-only assays detecting both free as well as bound ADAs against Infliximab or Adalimumab. The characteristics of these assays are described in Table 2.

### 2.4. Statistical Analysis

Results are presented as median [interquartile range (IQR)] or mean ± standard deviation, depending on Gaussian distribution. 

The paired samples *t*-test was used to compare paired Gaussian distributed biologic concentrations between assays. A *p*-value of <0.05 was regarded as statistically significant. 

Passing–Bablok regression, difference plots, and Spearman’s correlation analyses were performed to study the biologic concentration agreement between assays. A Spearman’s rho value of >0.7 was considered to be a highly positive correlation. 

Cohen’s kappa was used as a statistical measure to quantify the level of agreement between the Sanquin ADA assays and the Theradiag ADA assays. ADA concentrations of ≥12 AU/mL and ≥10 ng/mL were considered to indicate a positive result for the presence of ADAs for Sanquin and Theradiag, respectively. A value between 0 and 0.20 indicated “no agreement”, 0.21–0.39 indicated “minimal agreement”, 0.40–0.59 indicated “weak agreement”, 0.60–0.79 indicated “moderate agreement”, 0.80–0.90 indicated “strong agreement”, and ≥0.90 indicated “almost perfect” [10]. 

All statistical analyses were performed using GraphPad Prism (version 5.04, La Jolla, CA, USA) and R (version 4.4.1) using package ggplot2 (version 3.5.1). The R code is included as Supplementary Information.

## 3. Results

### 3.1. Patient Population 

The patients included in the distinct cohorts were from 42 ± 18 to 49 ± 18 years old and predominantly female. Most samples were collected from patients treated with a biologic by medical professionals in the gastrointestinal and liver diseases, rheumatology, ophthalmology, and pediatrics specialties (±94%). A minor proportion of the samples was collected from patients of the dermatology, pulmonology and plastic surgery departments (Table 3). 

### 3.2. Agreement between Infliximab and Adalimumab Concentration Assays

**Figure 1:**
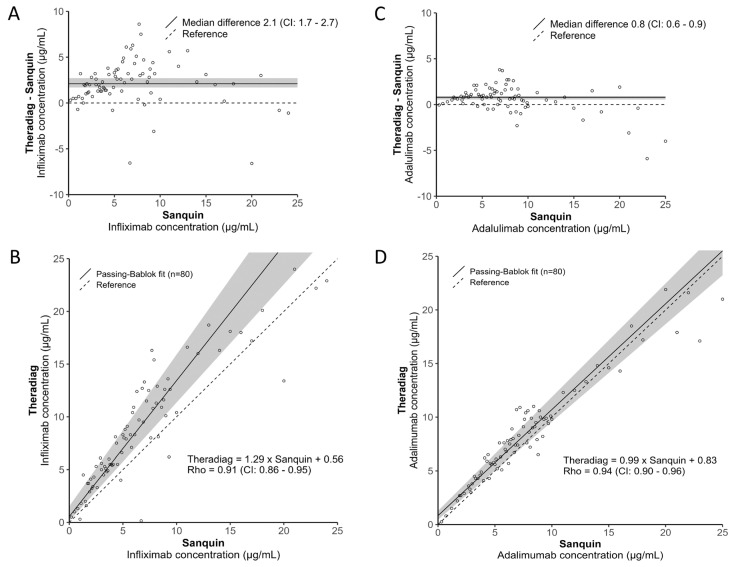
Infliximab (**A**) bias and (**B**) linear correlation and Adalimumab (**C**) bias and (**D**) linear correlation analyses between the Sanquin Diagnostics and Theradiag assays.

A broad spectrum of biologic concentrations ranging from 0.2 to 25 µg/mL, as based on the Sanquin assays, was studied, with a predominant focus on concentrations between 2 and 12 µg/mL (±75%). For Infliximab, the Sanquin median [IQR] concentration was 5.6 [3.1–8.5] µg/mL, while the Theradiag median [IQR] concentration was 8.1 [4.8–12.6] µg/mL (*p* < 0.001), revealing a median (CI) bias of 2.1 (1.7–2.6) µg/mL (Figure 1A). For Adalimumab, the Sanquin median [IQR] concentration was 6.8 [4.4–9.0] µg/mL, while the Theradiag median [IQR] concentration was 7.6 [5.3–10.1] µg/mL (*p* < 0.001), revealing a median (CI) bias of 0.8 (0.5–0.9) µg/mL (Figure 1C). Despite the statistically significant difference in biologic concentrations, there were strong positive correlations for both Infliximab (Spearman’s Rho = 0.91) and Adalimumab (Spearman’s Rho = 0.94) between the assays (Figure 1B,D).

### 3.3. Agreement between Infliximab and Adalimumab ADA Assays

The Theradiag ADA assays solely detecting free ADAs demonstrated weak to no agreement (Infliximab Cohen’s *k* = 0.458, Adalimumab Cohen’s *k* = 0.119), while there was moderate to strong agreement for the ADA assays detecting both free and bound ADAs (Infliximab Cohen’s *k* = 0.717, Adalimumab Cohen’s *k* = 0.802), when compared to the ADA assays of Sanquin (Table 4 and Table S1). Due to the limited sample availability, not all samples could be assessed for the research-only Theradiag ADA assays. The agreement difference was not explained by the difference in sample sets. The paired analyses demonstrated that the Infliximab Cohen’s *k* = 0.448 and Adalimumab Cohen’s *k* = 0.124. 

### 3.4. ADA Assay Discordance Analyses

Discordance analyses between the Sanquin ADA assay and the Theradiag ADA assay detecting solely free ADAs revealed median [IQR] biologic concentrations below their therapeutic windows, as measured using the biologic concentration assays of both manufacturers (Table 5). All samples for both biologics were positive for ADAs according to the Sanquin ADA assay, while ADAs were absent, as determined by the Theradiag ADA assay. Additionally, there was considerably more discordance observed for Adalimumab (*n* = 45) than for Infliximab (*n* = 16). 

Comparing the discordance between the Theradiag ADA assay, detecting both free and bound ADAs, and the Sanquin assay demonstrated only four discordant samples for each biologic (Table 6). For Infliximab, the biologic concentrations as measured with the assays of both manufacturers were more in the range of their therapeutic windows. The Sanquin ADA assay concluded that there was an absence of ADAs, while the Theradiag ADA assay determined the presence of ADAs in these samples. For Adalimumab, two discordant samples had decreased biologic concentrations while the other two samples had adequate concentrations. All discordant samples were positive for ADAs according to the Sanquin assay, while they were negative according to the Theradiag assay.

## 4. Discussion

Accurate therapeutic drug management, through assessing biologic concentration as well as ADA levels, is of pivotal importance when prescribing these medications. The present study investigated the biologic concentration and ADA assays of Theradiag and compared them to the assays of Sanquin Diagnostics in a patient population treated with Infliximab or Adalimumab. We report the following three major findings:

First, using a broad range of biologic concentrations, we demonstrated a strong correlation between the biologic concentration assays of Sanquin and Theradiag. This is in line with previous studies where the correlation between different methods has been shown to be relatively good [11]. However, there appears to be a significant bias in biologic concentration assessment, which was especially the case for Infliximab. This finding was also previously demonstrated by Berger et al. when comparing the CLIA (iTRACK10) to the ELISA (LISA-Tracker) tests of Theradiag, suggesting a methodological bias, amongst others [12]. Additionally, the bias is predominantly present for Infliximab, though concentration differences of more than 2 µg/mL between assays for multiple biologics have been previously observed [13]. However, as long as the same assay is used for follow-up, this restriction seems of limited clinical concern.

Second, the Theradiag ADA assays detecting solely free ADAs have weak to no agreement when compared to the ADA assay of Sanquin. From a technical perspective, this can, of course, be explained by the difference in analyte target. The absence of ADAs is measured by the Theradiag free ADA assays, but the presence of ADAs is measured by the Sanquin ADA assays, which demonstrated median biologic concentrations well below the therapeutic target. This suggests that the Sanquin ADA Theradiag free ADA assays do not detect relevant bound ADAs, causing decreased biologic concentration levels.

Third, the agreement between the Theradiag and Sanquin ADA assays detecting both free and bound ADAs is moderate to good. Antibodies are a highly variable mixture of molecules that are different in terms of epitope recognition, degree and type of glycosylation, isotype, and subclass distribution [14]. Additionally, each individual has their own antibody fingerprint, and the output of ADA assays depends on antibody characteristics, such as affinity and avidity, presented in non-uniform ways. All these factors prevent standardization and limit the comparability of results across assays [11,15].

### 4.1. Implications

The data presented in this retrospective assay comparison have important implications. Therapeutic drug management is heavily based on biological concentration and ADA assessment, in addition to clinical assessment. The observed correlation and agreement performance of the Theradiag biologic concentration and ADA (total) assays, as compared to the assays of Sanquin Diagnostics, grants the reliable use of Theradiag’s assays. Despite the Theradiag assays running on the automated platforms of Immunodiagnostic Systems (IDS), they cannot be combined with other tests and require system priming post-analyses. Nevertheless, implementing these assays would have practical advantages. The immunoassays have a faster turnaround time, are more suitable for high-throughput measurement, and require fewer technician resources. Also, this leads to enhanced therapeutic drug measurement, as sample collection, analysis and reporting, and an outpatient appointment can be scheduled on the same day. A point of attention is the observed bias in concentration assessment. Result interpretation should be carried out by taking the type of assay into account and will require careful communication with clinicians. Additionally, cost-effectiveness assessments will be required for clinical laboratories to investigate if the local patient population allows assay transition.

### 4.2. Limitations

This work is limited by the fact that selection criteria were used for the selection of patient samples which could lead to selection bias. However, the sample selection strategy that was applied minimized this bias. Second, the results in this study are only measured once. Nevertheless, the assays have been extensively validated by the manufacturers (intra and inter coefficients of variability (CVs) of approximately 5 and 10%, respectively), and quality controls were used, thereby diminishing this bias. Third, the current study’s results, for which only Theradiag ADA assays detecting both free and bound ADA were used, were not analyzed locally, but measured at Theradiag. However, the samples were anonymized and Theradiag did not have the results of the Theradiag free ADA and Sanquin ADA assays when analyzing these samples.

## 5. Conclusions

In conclusion, in this retrospective study, we demonstrated strong correlations and good agreement between the Theradiag and Sanquin Diagnostics assays measuring serum concentrations of Infliximab and Adalimumab and ADAs, both free and bound, against these biologics. The Theradiag ADA assay detecting solely free ADAs against Infliximab and Adalimumab had poor agreement with the assays of Sanquin Diagnostics when biologic concentrations were low, suggesting that these assays do not sufficiently detect clinically relevant ADAs. Despite good overall performance and correlations of the concentrations and ADA (total) assays, we recommend using the same assay format for long-term follow-up of patients treated with Infliximab and Adalimumab.

## Figures and Tables

**Table 1:** Characteristics of the Infliximab and Adalimumab concentration and ADA assays of Sanquin Diagnostics.

	Infliximab Concentration	Infliximab ADA	Adalimumab Concentration	Adalimumab ADA
Assay principle	ELISA	RIA	ELISA	RIA
Units	µg/mL	AU/mL	µg/mL	AU/mL
Cut-off	N.A.	<12 = negative 12–30 = ADA present, not quantifiable 30–875 = ADA present	N.A.	<12 = negative 12–30 = ADA present, not quantifiable 30–875 = ADA present
Measuring range	0.03–250	12–875	0.01–250	12–875
Ref. number *	J289	J288	J291	J290

ELISA = enzyme-linked immunosorbent assay, RIA = radioimmunoassay, AU = arbitrary units, ADA = anti-drug antibodies, N.A. = not applicable. * https://www.sanquin.org/nl/producten-en-diensten/diagnostiek/diagnostische-testen/index/name/ (assessed on 28 August 2024).

**Table 2:** Characteristics of the Infliximab and Adalimumab concentration and ADA assays of Theradiag.

	Infliximab Concentration	Infliximab ADA Free	Infliximab ADA Total	Adalimumab Concentration	Adalimumab ADA Free	Adalimumab ADA Total
Assay principle	CLIA					
Units	µg/mL	ng/mL	ng/mL	µg/mL	ng/mL	ng/mL
Cut-off	N.A.	10	10	N.A.	10	10
Measuring range	0.3–24	10–2000	10–2000	0.5–24	10–2000	10–2000
Ref. number	CTI 002–100	CTI 003–100	CTI 003T-50-R	CTA 002–100	CTA 003–100	CTA 003T-50-R

ADA = anti-drug antibodies, CLIA = chemiluminescence immunoassay, N.A. = not applicable.

**Table 3:** Baseline characteristics, including treating medical specialty, of the patients selected for biologic concentration and ADA agreement assessment.

	Infliximab Concentration	Infliximab ADA	Adalimumab Concentration	Adalimumab ADA
**Patient characteristics**				
*n*	80	80	80	79
Age (years)	42 ± 18	49 ± 18	48 ± 18	46 ± 18
Female (%)	56	73	56	65
**Medical specialty**				
Gastrointestinal and liver diseases (%)	68	48	74	68
Rheumatology (%)	10	28	5	9
Ophthalmology (%)	3	9	15	10
Pediatrics (%)	15	8	1	6
Internal medicine (%)	3	5	3	6
Dermatology (%)	0	4	0	0
Pulmonology (%)	3	0	0	0
Plastic surgery (%)	0	0	1	0
Other (%)	0	0	1	0

**Table 4:** Agreement analyses between the Sanquin Diagnostics ADA assays and Theradiag ADA (free and total) assays for Infliximab and Adalimumab.

Biologic	Theradiag ADA Assay	Number of Samples	Cohen’s *Kappa*
Infliximab	Free	80	0.458
Infliximab	Free (paired)	64	0.448
Infliximab	Total	64	0.717
Adalimumab	Free	79	0.119
Adalimumab	Free (paired)	74	0.124
Adalimumab	Total	74	0.802

**Table 5:** Discordance analyses of the Theradiag (free) vs. Sanquin Diagnostics assay.

		Sanquin Diagnostics		Theradiag	
Theradiag (Free) Assay	Number of Discordant Samples	Median [IQR] Biologic Concentration (µg/mL)	ADA Positive/Negative	Median [IQR] Biologic Concentration (µg/mL)	ADA Positive/Negative
Infliximab	16	1.3 [0.4–4.2] (*n* = 12)	All positive (*n* = 16)	1.4 [0.8–5.4] (*n* = 15)	All negative (*n* = 16)
Adalimumab	45	1.4 [0.6–3.9] (*n* = 43)	All positive (*n* = 45)	2.0 [0.5–5.3] (*n* = 41)	All negative (*n* = 45)

**Table 6:** Discordance analyses of the Theradiag (total) vs. Sanquin Diagnostics assay.

		Sanquin Diagnostics	Theradiag
Theradiag (Total) Assay	Number of Discordant Samples	Median [IQR] Biologic Concentration in µg/mL (and Corresponding ADA Result)	Median [IQR] Biologic Concentration in µg/mL (and Corresponding ADA Result)
Infliximab	4	2.6 (negative)	3.2 (positive)
		3.3 (negative)	3.3 (positive)
		5.1 (negative)	4.6 (positive)
		7.8 (negative)	9.4 (positive)
Adalimumab	4	0.2 (positive)	<0.5 (negative)
		0.3 (positive)	<0.5 (negative)
		4.1 (positive)	5.7 (negative)
		4.7 (positive)	7.9 (negative)

## Data Availability

The original contributions presented in the study are included in the article/Supplementary Materials; further inquiries can be directed to the corresponding author/s.

## Supplementary Materials

The following supporting information can be downloaded at: https://www.mdpi.com/article/10.3390/antib13030073/s1, Table S1: Detailed agreement analyses between the Sanquin Diagnostics ADA assays and Theradiag ADA (free and total) assays for Infliximab and Adalimumab.
