# Supplementary material for: Therapeutic Drug Monitoring of Infliximab and Adalimumab through Concentration and Anti-Drug Antibodies Assessment; Comparison of Sanquin Diagnostics and Theradiag Assays
Source: Antibodies (Basel). 2024 Sep 5;13(3):73. doi: 10.3390/antib13030073 (PMC11417797; doi:10.3390/antib13030073)
Supplement: Supplementary file 1 [file antibodies-13-00073-s001.zip › antibodies-3145937-supplementary.pdf]

Supplementary Table S1. Detailed agreement analyses between the Sanquin Diagnostics ADA assays and Theradiag ADA (free & total) assays for Infliximab and Adalimumab.

		Sanquin	
	**Infliximab ADA (n=80)**	positive	negative
Theradiag free	positive	54	0
	negative	16	10
		Cohen's kappa	0.458

		Sanquin	
	**Infliximab ADA (n=64)**	positive	negative
Theradiag free	positive	39	0
	negative	15	10
		Cohen's kappa	0.448

		Sanquin	
	**Infliximab ADA (n=64)**	positive	negative
Theradiag total	positive	54	4
	negative	0	6
		Cohen's kappa	0.717

		Sanquin	
	**Adalimumab ADA (n=79)**	positive	negative
Theradiag free	positive	24	0
	negative	45	10
		Cohen's kappa	0.119

		Sanquin	
	**Adalimumab ADA (n=74)**	positive	negative
Theradiag free	positive	22	0
	negative	42	10
		Cohen's kappa	0.124

		Sanquin	
	**Adalimumab ADA (n=74)**	positive	negative
Theradiag total	positive	60	0
	negative	4	10
		Cohen's kappa	0.802
